# Substance Abuse, Depression, and Social Anxiety: Case Study and Application of Cognitive Psychotherapy

**DOI:** 10.1155/2023/3430636

**Published:** 2023-08-08

**Authors:** Feda Abu Al-Khair

**Affiliations:** Assistant Professor in Clinical Psychology, Al-Ahliyya Amman University, 19328, Amman, Jordan

## Abstract

A 20-year-old male was referred by a psychiatrist to the clinic for treatment. He was diagnosed with social anxiety disorder (SAD), depression, and substances abuse. He complained of depressive mood and severe anxiety symptoms. These symptoms are triggered in social situations, as well as when talking to others, being in public areas, and going to malls or any crowded places. Because of his symptoms, he avoided getting into the situation, which affected his daily life. The patient was diagnosed with SAD, major depression, and substance abuse and underwent 20 separate sessions of cognitive behavioral therapy (CBT). The application of CBT led to a decrease in the number of anxiety attacks and angry outbursts that the patient suffers from. It also helped him learn some techniques to use in his interactions within the society, as well as other techniques, such as cognitive reorganization of dysfunctional thoughts, and gradually exposed him to the social situations. He also learned to practice some relaxation techniques, to teach him integration in social situations and confrontation instead of avoidance.

## 1. Introduction

Social anxiety disorder (SAD), one of the most common anxiety disorders, is rapidly increasing and disproportionately affects young people [1]. It is the third most common mental disorder after depression and alcohol dependence. According to epidemiological reports, the current prevalence is 5%–10%, and the lifetime prevalence is 8.4%–15% [2]. However, based on prevalence studies in seven countries, researchers found that the global prevalence of social anxiety is significantly higher than previously reported, and more than one-third of respondents met the threshold for SAD Standard. If left untreated, it can cause many serious problems in people's lives [1, 3].

People with SAD experience significant fear and/or anxiety, in one or more types of situations on the social level, where they think others will judge and negatively evaluate them. The danger they feel during these situations is excessive compared to the feelings of a regular person, in form and intensity, when the two are placed in the same sociocultural context [4].

Comorbidity factors: SAD commonly occurs alongside other anxiety disorders, major depressive disorder, and substance use disorders. Typically, SAD emerges before the onset of these other disorders, except for specific phobia and separation anxiety disorder. Prolonged social isolation resulting from SAD can lead to the development of major depressive disorder. Among older adults, there is a significant comorbidity between SAD and depression. Individuals may turn to substances as a form of self-medication to cope with social fears. However, the symptoms of substance intoxication or withdrawal, such as trembling, can also intensify social anxiety. Body dysmorphic disorder frequently coexists with SAD, and generalized SAD often co-occurs with avoidant personality disorder. In children, there is a high prevalence of comorbidities between SAD and high-functioning autism spectrum disorder, as well as selective mutism [4].

A review highlighted the significance of the relationship between alcohol use and individuals suffering from SAD who believe that consuming alcoholic beverages will have a positive effect on them. People suffering from disorders, such as SAD and alcohol use disorder (AUD), expect alcohol to have more positive effects in social settings (such as decreased tension and social assertiveness) than those not suffering from AUD and those with lower levels of anxiety [5–7].

Many studies have been conducted on cannabis, nicotine, and other dependencies; however, many others concentrate on alcohol, when investigating the co-occurrence of alcohol and substance use disorder (ASUD) and SAD [8]. It is widely acknowledged that people with SAD will more likely be the consumers of alcoholic beverages and/or abuse drugs. Comorbid SAD and ASUD are clinically significant because they are linked to higher morbidity, poorer treatment outcomes, and decreased therapy seeking.

People with anxiety conditions are typically advised to begin using drugs (self-medication) to treat their symptoms. However, due to alterations in the biology of the brain, substance use can cause or contribute to a person's susceptibility to suffer from anxiety [5, 6, 9].

The study aims to determine the efficacy of cognitive psychotherapy in cases of SAD, depression, and substance abuse. Cognitive behavioral therapy (CBT) should have a major impact in these cases, by reducing the patient's complaints, such as anxiety from speaking in public and from social situations, low mood, and symptoms of depression. It should also allow patients to return to their studies and social life, without having to depend on different substances.

### 1.1. Description of Case Study and Methods

#### 1.1.1. Case Report

The patient is a 20-year-old single man. He is unemployed and has a history of polysubstance abuse and family problems. For the past 2 years, he has used alcohol, cocaine, and medical substance (Lyrica and other medications). He has a history of social anxiety with biological symptoms, which are unresponsive to medication. He expressed low self esteem and feelings of worthlessness. He was pessimistic about the future, saying, “I don't see anything ahead for me.” He described a passive death wish, but denied having any active suicidal thoughts. He described feeling fast heartbeats, blushing, trembling, sweating, trouble catching his breath, dizziness, and panic complaints, especially when talking to people and women in specific. He also described being unable to carry out a conversation with others, feelings of being watched and laughed at, and believing that he is ugly looking. He described having these complaints since adolescence. The patient has been an addict for the past 2 years. He started with Marijuana and alcohol, and then he tried several medical drugs.

The patient talked about various upsetting memories of instances that took place during his childhood and up to his late teenage years. This included memories, such as not being given a choice or participating in decision making, his father's absence from the house due to travel, followed by the discovery of his marriage to another woman and the presence of children. His family's upbringing style is firm, authoritarian on the part of the mother, and permissive on the part of the father.

### 1.2. Assessment and Diagnosis

Depending on psychiatrist referral letter, Structured Clinical Interview and Several tools were used to evaluate and diagnose patient's social anxiety and depression symptoms. They varied from self to clinician administered measures, and included the following:

#### 1.2.1. Beck Depression Inventory (BDI)

The BDI [10] is constituted of 21 items. It is self-administered and represents an assessment of the physiological, affective, and cognitive aspects of depression, and is a measure of its severity. A total score of 10 or less is considered normal. A person is considered clinically depressed, if he or she obtains a score of 20 or more, on the BDI. The BDI is characterized by its high reliability and validity. Treatment outcome research makes use of the characteristics of this scale. The scale was translated by Abdul Khaleq 1996, and it has high reliability and validity in the Arab regions, as stated in [11–14].

#### 1.2.2. Liebowitz Social Anxiety Scale (LSAS)

Twenty-four items constitute the LSAS [15]. It is administered by a clinician, so that the respondent may rate his or her feelings of fear and avoidance, on a scale of 0 (none) to 4 (extreme). It was translated to Arabic by Ibrahim [16], and it has high reliability and validity in the Arab regions, as stated in [17, 18].

To examine SAD, the LSAS, with its good psychometric properties, is often employed in treatment outcome research [19]. Cutoff scores determined by Mennin et al. [20] for social phobia are greater than 30 and greater than 60 for generalized social phobia.

### 1.3. Formulation

Based on the assessment of the patient's case, which was determined from the psychiatrist's letter and the information he provided during the first meeting, he was diagnosed with social anxiety and depression. The patient showed symptoms of social anxiety, which were concluded to be the signs of his low self-esteem, manifested through avoidance. Depression was also another type of manifestation he exhibited, with an experience of persistent low spirits, ruminations about the past, and feeling guilty for exhibiting angry outbursts during current times. As a result, while patient's symptoms satisfied the diagnosis for social anxiety, they seemed to result from his depression, which started during adolescence. The CBT longitudinal model [21] was thought to be the best tool to use to understand patient's depression in view of the experiences he had in earlier life. These are the core beliefs, negative in nature, he has derived in life, as well as the rigid life rules, all of which contributed to his low self-esteem and led him to demonstrate signs of depression. Social anxiety has led him to drug addiction, due to his beliefs that the drug's effects will encourage him to talk to and deal with people. It is worth noting that the patient has been undergoing pharmacological treatment with his psychiatrist since the beginning.

### 1.4. Therapy Program

Fennell's [22] guidelines were followed for all sessions:

- Establishing the agenda.
- Reviewing events that have taken place since the previous session, feedback on the previous session, and homework.
- Going over the agenda once more.
- Prioritizing and discussing agenda items.
- Collaboratively assigning homework.
- Checking reactions.

### 1.5. Techniques of Cognitive Behavioral Therapy

#### 1.5.1. Self-Monitoring

Self-monitoring refers to observing one's behaviors and experiences systematically during various occasions for a certain time.

It is used in the therapy as a method of intervention, as it helps patients examine thoughts, emotions, and behaviors. It helps them identify the situations they are afraid of and find the best course of action for dealing with them.

Drastic changes occur from self-monitoring, according to Kazdin [23]. Korotitsch and Nelson-Gray [24] concluded that an immediate change is among the therapeutic effects of self-monitoring, despite their small scale. The clinician required the patient to track his thoughts, feelings, behaviors, as well as any differences he notices in himself.

#### 1.5.2. Cognitive Reorganization

The four stages of cognitive reorganization, according to Beck et al. [25] are: (i) identifying dysfunctional thoughts, (ii) cognitive reorganization, (iii) modifying dysfunctional thoughts, and (iv) assimilating the new functional thoughts. On the second stage, patients begin to recognize their automatic or dysfunctional thoughts, as well as the emotions associated with them. For instance, a recurring thought for the patient was that others see him as an ugly, insignificant person. This notion contributed to his feelings of anxiety and fear.

Nevertheless, another adaptive thought that he can adopt instead is: “I may not like everyone, but there are those who love me as I am”.

As a result, the patient was taught during all sessions to use adaptive thoughts instead of negative thoughts. Part of six of his sessions was dedicated to discussing a record he kept of his dysfunctional thoughts.

#### 1.5.3. Relaxation

Relaxation techniques were used to treat a patient's symptoms, particularly those that resulted from his anxiety and depression and were physiological in nature.

According to Jacobson's technique, there are certain breathing and muscle relaxation exercises in use, which can be beneficial in a patient's case. As such, the clinician worked on teaching him these exercises over eight sessions, to help him manage the physical symptoms he suffers from. The patient was taught to use deep breathing and some short muscle relaxation techniques in his day-to-day life, particularly when confronted with an unpleasant situation [26].

#### 1.5.4. Training in Assertiveness and Motivation

Assertiveness training can be beneficial in cases where the patients suffer from depression, anxiety in social settings, addiction, and issues related to unspoken anger.

Since it is now known that assertiveness is learned rather than an inborn trait, assertiveness training can be employed in enhancing self-esteem and ameliorating interpersonal skills. It is true that some people seem more assertive, nevertheless, assertiveness can be acquired. In the patient's case, he was assisted in determining the situations where he faces more challenges on an interpersonal level, as well as the behaviors he exhibits that he needs to concentrate on in order to improve. Furthermore, the therapist assisted him in identifying the beliefs and attitudes that he may have developed, which caused him to become too passive. As part of this technique, she used role-playing exercises.

#### 1.5.5. Clinical Sessions

The patient had one-on-one sessions for 50 min with his therapist each to complete his therapeutic treatment, over the course of 5 months. The reasons for employing CBT were examined during the first session. Educating the patient on SAD, depression, and addiction were a point of focus during therapy. Automatic thoughts and how they affect cognition were examined, helping him in identifying these types of thoughts and feelings as he experienced them.

As homework, he was given an anxiety self-monitoring diary. The therapist emphasized the issue of establishing and maintaining a good relationship between patient and clinician during the course of therapy. The patient described certain situations, as well as important events in his life where he felt that his symptoms were worse. This took place during the second session, where he also got the diary for dysfunctional thoughts as homework, after going through the explanation about cognitive reorganization and its four stages. On the third session, he learned breathing and muscle relaxation exercises, from a specialist, to acquire tools to help him relax and effectively manage his stress. Eight 20-min sessions were followed with similar exercises. He was also asked to both practice these sessions at home and track his progress on a daily basis.

From session four to session nine, time was devoted to using adaptive responses to challenge dysfunctional thoughts. Initially, there was an attempt to identify automatic negative thoughts in certain situations, during which he was asked to keep track of his moods. After recognizing patient's negative thoughts, emotions, and behaviors, the work was done to verify the evidence that supported them.

To help the patient effectively socialize with others, sessions 10–12 were dedicated to learning assertiveness skills. The therapist talked to the patient about the meaning of assertiveness for him, the reasons that prevented him from becoming assertive, and differences in behaviors, from assertiveness to aggressiveness, passing by submissiveness. This information has proved useful to him. To practice such skills, the exercises incorporating roleplay were used.

During the following sessions (13–20), situations that induce anxiety in the patient were investigated and then ordered according to the level of anxiety he feels during each. Through exposure techniques, the patient was confronted with each level of anxiety in real time, where he got to practice each step, until he signaled his confidence to proceed to the level to follow.

The last session saw the patient discussing how he got over difficult situations, including being introduced to people for the first time, visiting friends, public speaking, and participating in presentations in class. He was able to challenge his cognition in the situations he had previously identified as difficult and then employed the techniques he learned (breathing and muscle relaxation) to manage symptoms of anxiety.

Preventing relapse was discussed during the last session, as well as methods to achieve this purpose, and other methods for surmounting the difficulties and failures of the past. Finally, there was a discussion related to how to apply change to the skills he learned and how to use the new techniques he acquired on a day-to-day basis.

## 2. Results and Discussion

It was clearly seen that patient's levels of anxiety in daily social situations were effectively improved through CBT, in case of SAD. According to the exposure sessions, which also included a discussion of patient's efforts outside of the clinical setting, The patient had the courage to walk through a pedestrian crossing and look at people, ask a security guard for directions, and communicate with new people in public places.

Findings show that CBT helped the patient reduce his anger, cravings, and stress (see Table 1). Furthermore, it improved his sleep quality and assertiveness. These findings are consistent with the previous research results. Cannabis use disorder was effectively treated with CBT, a treatment method which was rendered even more effective with the use of medication. A patient's functions (physiological, psychosocial, and social) are affected by substance use and its related disorders, but CBT significantly aids in the treatment of deficits at these levels [27, 28].

It was found that when treating alcohol and opioid withdrawal disorders in a rehabilitation facility, CBT was effective. Patients felt more at ease staying in therapeutic sessions, after the detoxification process. A previous study found that best results could be obtained from a combination of pharmacotherapy and CBT, during the treatment of patients suffering from substance use disorder.

During the current study, CBT sessions, organized, structured, and running on an individual basis between patient and therapist were conducted. Each session had a planned objective and a set agenda. The patient's cognitive reorganization, stress management, daily living functions, and lapse relapse prevention were all prioritized. The patient's depression and social anxiety significantly improved, because the results on the scales have been decreased (see Table 2).

The therapeutic process of the patient investigated in this study, suffering from substance use disorder, included “skills training”, a technique also known as “skills building”. Much focus was placed on any deficits, the patients suffer from related to emotions, cognition, behaviors, organization, problem solving, and interpersonal relations, during skill building.

The treatment made use of any approach targeting individual differences between patients. The connections the patient developed with others were a point of focus because the opposite of addiction is not sobriety, but rather connection with society. The patient's interpersonal skills were also targeted in this study. Such skills aid in the resolution of relationship complications. They help enhance effective communication and allow the patient to make use of it along with social support. Assistance received from all these tools helps individuals abstain from addictive materials and promotes the establishment of healthy relationships [29].

## 3. Conclusion

CBT was effective in decreasing the patient's symptoms, including his low moods, avoidance of social interactions, and anger. It also affected the patient in terms of his gradual return to work and positive relationships with his family members.

## Figures and Tables

**Table 1:** Thoughts record.

Situation	Mood	Automatic negative thoughts	Evidence that supports the negative thoughts	Evidence that does not support the negative thoughts	Alternative/balanced thoughts	Mood
—	—	—	—	—	—	—

**Table 2:** Outcome measures for social anxiety and depression.

Outcome measures	Referral and assessment (18/07/2022)	Session 7 (29/08/2022)	Session 13 (10/10/2022)	Session 20 (28/11/2022)
BDI	20 (moderate)	16 (moderate)	14 (mildly moderate)	11 (mild)
LSAS	50 (moderately severe)	47 (moderate)	45 (moderate)	40 (moderate)

## Data Availability

The data used to support the findings of this study have not been made available because of the privacy issues.

## Additional Points

*Study Limitations*. The patient had fluctuations in motivation. The family has gone through a lot of stressful life events. The patient's father is busy and does not frequently communicate with him. The study approach was cognitive behavioral therapy (CBT), and the case had multiple problems from childhood. Perhaps it would be more useful to use some analytical or Gestalt techniques to deal with past problems.
